# RPE phagocytic function declines in age-related macular degeneration and is rescued by human umbilical tissue derived cells

**DOI:** 10.1186/s12967-018-1434-6

**Published:** 2018-03-13

**Authors:** George Inana, Christopher Murat, Weijun An, Xiang Yao, Ian R. Harris, Jing Cao

**Affiliations:** 1 Department of Ophthalmology, Bascom Palmer Eye Institute, University of Miami School of Medicine, 1638 N.W. 10th Avenue, Miami, FL 33136 USA; 2 Janssen Research & Development, LLC, San Diego, CA 92121 USA; 3 Janssen Research & Development, LLC, Spring House, PA 19477 USA

**Keywords:** Age-related macular degeneration, Cell therapy, Human umbilical tissue derived cells, Retinal pigment epithelium, Phagocytosis, Receptor tyrosine kinase, Bridge molecules

## Abstract

**Background:**

Age-related macular degeneration (AMD) is a leading cause of blindness among the elderly characterized by retinal pigment epithelium (RPE) degeneration with accumulation of abnormal intracellular deposits (lipofuscin) and photoreceptor death. RPE is vital for the retina and integrity of photoreceptors through its phagocytic function which is closely linked to formation of lipofuscin through daily phagocytosis of discarded photoreceptor outer segments (POS). Although phagocytosis has been implicated in AMD, it has not been directly shown to be altered in AMD. RPE phagocytic defect was previously shown to be rescued by subretinal injection of human umbilical tissue derived cells (hUTC) in a rodent model of retinal degeneration (RCS rat) through receptor tyrosine kinase (RTK) ligands and bridge molecules. Here, we examined RPE phagocytic function directly in the RPE from AMD patients and the ability and mechanisms of hUTC to affect phagocytosis in the human RPE.

**Methods:**

Human RPE was isolated from the post-mortem eyes of normal and AMD-affected subjects and cultured. RPE phagocytic function was measured in vitro using isolated POS. The effects of hUTC conditioned media, recombinant RTK ligands brain-derived neurotrophic factor (BDNF), hepatocyte growth factor (HGF), and glial cell-derived neurotrophic factor (GDNF), as well as bridge molecules milk-fat-globule-EGF-factor 8 (MFG-E8), thrombospondin (TSP)-1, and TSP-2 on phagocytosis were also examined in phagocytosis assays using isolated POS. RNA was isolated from normal and AMD RPE treated with hUTC conditioned media and subjected to transcriptome profiling by RNA-Seq and computational analyses.

**Results:**

RPE phagocytosis, while showing a moderate decline with age, was significantly reduced in AMD RPE, more than expected for age. hUTC conditioned media stimulated phagocytosis in the normal human RPE and significantly rescued the phagocytic dysfunction in the AMD RPE. RTK ligands and bridge molecules duplicated the rescue effect. Moreover, multiple molecular pathways involving phagocytosis, apoptosis, oxidative stress, inflammation, immune activation, and cholesterol transport were affected by hUTC in the RPE.

**Conclusions:**

We demonstrated for the first time RPE phagocytic dysfunction in AMD, highlighting its likely importance in AMD, and the ability of hUTC to correct this dysfunction, providing insights into the therapeutic potential of hUTC for AMD.

**Electronic supplementary material:**

The online version of this article (10.1186/s12967-018-1434-6) contains supplementary material, which is available to authorized users.

## Background

Age-related macular degeneration (AMD) is a major cause of blindness for the older population (> 60 year old) in the western world [1–4]. The part of the retina responsible for central vision (macula) is mainly affected, resulting in loss of vision for recognizing faces, driving, and reading. Thus, it is a devastating disease for individuals who are still physically active, limiting their everyday activities significantly. The retinal pigment epithelium (RPE), a monolayer of cells behind the light-sensing photoreceptors in the retina, is the primary target for destruction in AMD [5]. There are two forms of the disease, the atrophic (dry) and the exudative (wet) [2]. The atrophic form is characterized by regional pattern of RPE degeneration called geographic atrophy whereas the exudative form shows neovascularization from the choroid underneath the RPE called choroidal neovascularization [2, 3]. The vast majority of patients with AMD have the atrophic form. Although the atrophic form of the disease is much more prevalent, the largest numbers of patients that develop severe visual loss have the exudative form of AMD [6–8]. Among Caucasians over 40 years old the incidence of AMD is reported to be ~ 6.8% for early AMD and ~ 1.5% for late AMD [6], and the incidence is increasing rapidly, predicted to double by 2050 [6–8]. Currently, treatment for AMD is only available for the wet form of the disease, with anti-VEGF drugs being the standard care but not a cure. There are no effective treatments for dry AMD [9–12]. Meanwhile, with increasing incidence of dry AMD which can relentlessly progress to blindness over time, an effective treatment is urgently needed.

Scientists have been in pursuit of the cause or causes of AMD for a century, but the exact etiology of this disease is not known yet. Hallmarks of the disease include accumulation of abnormal deposits inside (lipofuscin) and outside (drusen) the RPE and slow degeneration of the RPE [5, 13–18]. In general AMD is thought to be caused by a combination of genetic and environmental factors [1–3]. Pathogenic mechanisms proposed for AMD include oxidative stress, inflammation, and immune activation, all centering around the RPE [5, 19–24]. Besides being the blood-retinal barrier, RPE performs many functions crucial for retinal preservation and vision. An important one is to phagocytize the daily-shed photoreceptor outer segments (POS), which is critical for photoreceptor survival and function and, thus, vision [5, 25]. A key connection between the proposed pathogenic mechanisms of AMD and the RPE is the mentioned hallmarks, lipofuscin and drusen. Phagocytosis of POS, one of the key functions of the RPE, has an important relationship to lipofuscin, a key factor in the pathogenic mechanism of AMD, because many of its components derive from material phagocytized by the RPE [26]. Lipofuscin, shown to build up in the RPE with age, is an incomplete degradation product of lipids and proteins, intimately related to oxidative stress in both cause and effect [1, 27–29]. Its components, including the bis-retinoid A2E, are toxic to the cell, and are postulated by some to lead to RPE death in AMD [30, 31]. Drusen, somewhat similar in composition to lipofuscin, accumulates below the RPE and, in addition to presenting a physical barrier to RPE exchange function, shows evidence of involvement with inflammation and immune activation that are associated with RPE damage and death in AMD [17, 18, 32–36]. The decrease in RPE phagocytic function with aging has been postulated to lead to drusen deposition and increased lipofuscin accumulation, resulting in RPE death and AMD [19, 24, 37, 38]. A2E in lipofuscin and increased iron level in RPE have been shown to inhibit RPE phagocytosis, leading to RPE death and AMD [3, 39–42]. Therefore, phagocytosis has been demonstrated or postulated to be involved in the pathogenesis of AMD both in cause and effect. In support of the importance of RPE phagocytosis for AMD, animal models that have created a defect in phagocytosis, namely the RCS rat, the alphavBeta5 knockout, and the cathepsin D transgenic, all have shown retinal degeneration, and phenotypes resembling AMD were seen in the latter two [43–45]. In all of these cases, a dysfunction in RPE phagocytosis is present. However, a dysfunction in RPE phagocytosis has not been actually demonstrated in AMD.

We showed before that human umbilical tissue-derived cells (hUTC) and media conditioned by these cells (hUTC CM) can correct the defect in RPE phagocytosis present in the RCS rat model of retinal degeneration through the secretion by hUTC of RTK ligands and bridge molecules [46]. Demonstration of the capacity of hUTC to rescue RPE phagocytic dysfunction in the RCS model and the elucidation of the mechanism by which hUTC does this encouraged us to investigate its potential in other retinal degenerative conditions. Thus, in this work we studied POS phagocytosis by RPE from AMD and normal donors with varying age, the effect of hUTC on RPE phagocytosis, and the mechanisms involved. Our findings showed that RPE phagocytosis decreased moderately with age in normal donors, but significantly declines in the RPE from individuals with AMD compared to normal. hUTC was effective in correcting the phagocytic dysfunction in the AMD RPE. The effect of hUTC on phagocytosis rescue was mimicked by RTK ligands brain-derived neurotrophic factor (BDNF), hepatocyte growth factor (HGF), and glial cell-derived neurotrophic factor (GDNF), as well as opsonizing bridge molecules milk-fat-globule-EGF-factor 8 (MFG-E8), thrombospondin (TSP)-1, and TSP-2. Moreover, using RNA-Seq transcriptomic gene profiling, we identified multiple cellular and biological processes that were affected by hUTC treatment and could be associated with the phagocytosis rescue effect of hUTC.

## Methods

### Cells and cell culture

#### Human primary RPE cell isolation and culture

The study was approved by the institutional ethics committee and was conducted in concordance with the tenets of the Declaration of Helsinki. Primary retinal pigment epithelial cultures were established from noninfectious human cadaver eyes from donors with no known ocular diseases or with AMD confirmed by our ocular pathologists that were obtained from certified eye banks (Florida Lions Eye Bank, Bascom Palmer Eye Institute, Miami, FL; San Diego Eye Bank, San Diego, CA; National Disease Research Interchange (NDRI), Philadelphia, PA). The human globe, received in moist chamber (within 60 h of death), was rinsed in PBS with penicillin 200 U/ml/streptomycin 200 μg/ml (Life Technologies, Carlsbad, CA), and the anterior segment was removed at the limbus. After gross examination and photography, the retina was removed, and the posterior pole was fragmented and digested in 4 ml of 2% (w/v) Dispase solution (Roche Diagnostics) for 5 min at 37 °C. After terminating the digestion with excess media (Dulbecco’s modified Eagle’s medium (DMEM) (Life Technologies), 25 mM HEPES (Sigma-Aldrich), 200 U/ml Pen-Strep) the tissues were allowed to incubate in fresh media for 6–12 h at 37 °C. The RPE cells were teased off the tissues in RPMI media (Life Technologies), rinsed with HBSS (Life Technologies), resuspended in 0.5 ml of 0.1% (w/v) Trypsin, pH 8.0 (Life Technologies), and incubated for 2 min at 37 °C. The RPE cells were dispersed by trituration, rinsed in excess Minimum Essential Medium (MEM)/20% (v/v) FBS (Life Technologies), and centrifuged at 1000×*g*. The RPE cells were resuspended in appropriate volumes of MEM/20, cultured on glass cover slips in 24-well plates using a culturing cylinder or tissue culture flasks, and used within 5 passages from the original culture. The cultured RPE cells were confirmed to be RPE by immunostaining with the Pan-Keratin (C11) monoclonal antibody (Cell Signaling Technology, Inc. Danvers, MA) (Additional file 1: Fig. S1) [47].

#### Immunofluorescence of RPE cells

RPE cells fixed with 4% paraformaldehyde in 24-well plate wells were washed with PBS, blocked with 1% BSA (Sigma-Aldrich Cat#A-7030) + 10% newborn goat serum (Life Technologies, Cat#16210-064) + 0.1% TritonX-100 (Sigma-Aldrich, Cat#T-8787) in PBS for 1 h, then incubated with the primary antibody (Pan-Keratin C11 Mouse mAb, Cell Signaling, Catalog #4545S, 1:400 dilution with PBS containing 1% BSA) for 2 h at RT, washed, and finally with Alexa Green-conjugated secondary antibody (1:50 dilution with PBS, 1 h at RT) (Life Technologies, Catalog #A21202, Eugene, OR). The control consisted of treatment with only the secondary antibody after blocking. After final washing, the preparations were mounted in Vectashield mounting fluid (VWR, Cat#101098-042) and examined under fluorescence in Photomicroscope III equipped with a digital camera.

#### hUTC isolation and culture

Human umbilical cords were obtained with donor consent following live births from the National Disease Research Interchange (Philadelphia, PA). Tissues were minced and enzymatically digested. After almost complete digestion with a Dulbecco’s modified Eagle’s medium (DMEM)-low glucose (Lg) (Invitrogen, Carlsbad, CA) containing a mixture of 0.5 U/ml collagenase (Sigma, St. Louis), 5 U/ml neutral protease (Serva Electrophoresis, Heidelberg Germany), and 2 U/ml hyaluronidase (Cumulase, Origio), the cell suspension was filtered through a 70 µm filter, and the supernatant was centrifuged at 250×*g*. Isolated cells were washed in DMEM-Lg three times and seeded at a density of 5000 cells/cm^2^ in DMEM-Lg medium containing 15% (v/v) FBS (Hyclone, Logan, Utah). When cells reached approximately 70% confluence, they were passaged using TrypLE (Gibco, Grand Island, NY). Cells were harvested after two to three passages and banked.

### Isolation of POS from human eyes and phagocytosis assay

Isolation of POS and phagocytosis assay were performed as described before [48]. Retinas from human eyes were isolated, homogenized with Polytron (5 mm generator), and fractionated by 27–50% (w/v) linear sucrose gradient centrifugation at 240,000×*g* for 1 h at 4 °C. The POS bands (up to 3 bands) which represent homogenized POS particles of different sizes were collected, diluted with HBSS, centrifuged at 8000×*g*, and resuspended in serum-free culture medium. The FITC stock solution (2 mg/ml in 0.1 M sodium bicarbonate, pH 9.0–9.5) (Sigma, St. Louis) was added to a final concentration of 10 μg/ml and incubated at room temperature for 4 h. The FITC-stained POS was pelleted by centrifugation, resuspended in MEM containing 20% (v/v) FBS, and stored at 4 °C for experiment use.

For phagocytosis assay, 5 × 10^4^ human RPE cells are plated out on a circular glass cover slip in each of the wells in a 24-well plate using a culturing cylinder (0.38 cm^2^ area, 1.32 × 10^5^ cells/cm^2^), maintained in MEM containing 20% (v/v) FBS for at least 6 days, then in MEM with 5% (v/v) FBS before the assay. The assay is started by overlaying the culture with FITC-POS (0.5–5 × 10^6^) and incubating at 37 °C for 8–12 h. At the end of the incubation, the cells are vigorously washed with PBS to remove uningested POS and fixed with 2% (w/v) paraformaldehyde. The ingested POS were visualized by fluorescence microscopy at ×250 magnification with Zeiss fluorescence Photomicroscope III. Phagocytosis level is quantitated microscopically by counting the number of internalized fluorescent POS in 10 representative fields (field size, 0.021 mm^2^) and averaging them as described before [46, 48]. The ingested POS are clearly distinguishable as small, mostly circular, sharply demarcated, fluorescent particles, intracellular in distribution.

### RNA-Seq and gene analysis

Cells isolated from normal or AMD donors were seeded in 24-well plates at 5 × 10^4^ cells per well in MEM containing 20% FBS and cultured for at least 1 week. Then some of the cells were subjected to medium change to hUTC CM or control medium. These cells were cultured for another 24 h. The cells before medium change and the cells after medium change and culture were collected for total RNA extraction and DNA removal using the Qiagen RNAeasy extraction and on-column DNAse kit (Qiagen, Valencia, CA). The integrity and quantity of RNA in the samples was determined using the NanoDrop 1000 spectrophotometer (Thermo Fisher Scientific, Waltham, MA) and Agilent 2100 Bioanalyzer (Agilent Technologies, Santa Clara, CA). Library preparation and sequencing were performed by Q2 Solutions (Morrisville, NC). RNA libraries were prepared using Illumina’s TruSeq total RNA Sample Prep kit following the manufacturer’s instructions, and sequenced with Illumina’s HiSeq 2000. All RNA sequencing data were processed using ArrayStudio tool (Omicsoft, Cary, NC). Human sequence reads were mapped onto genome assembly GRCh38 (http://www.ncbi.nlm.nih.gov/projects/genome/assembly/grc/human/).

refGene gene model (http://genome.ucsc.edu/, data created on 2015-07-18) was applied to represent genes in human genomes. TPM (Transcripts Per Million) was applied to calculate gene expression in each sample and to normalize across samples. Gene Ontology (http://geneontology.org/) and Gene Set Enrichment Analysis (GSEA) using Ingenuity Pathway Analysis (IPA) tool (Qiagen, Redwood City, CA), were applied for gene analysis and classification.

### Preparation of hUTC conditioned medium

hUTC were cultured in DMEM low glucose medium containing 15% (v/v) FBS (Hyclone, Logan, Utah) and 4 mM l-glutamine (Gibco, Grand Island, NY) for 24 h in 37 °C 5% CO_2_ incubator. Medium was aspirated and replenished with DMEM:F12 (American Type Culture Collection (ATCC), Manassas, VA) medium containing 10% (v/v) FBS and cultured for another 48 h. Control medium (DMEM:F12 medium containing 10% (v/v) FBS) was also cultured for 48 h in parallel. The cell culture supernatant and control medium were collected and centrifuged at 250×*g*, 5 min at 4 °C. The supernatants were aliquoted and frozen immediately at − 70 °C until use.

### Human recombinant proteins

Recombinant human BDNF, GDF, MFG-E8, TSP-1 and TSP-2 (R&D Systems, Inc. Minneapolis, MN). Recombinant human HGF (Life Technologies Carlsbad, CA).

### Statistical analysis

Statistical significance for phagocytosis assay results was assessed by unpaired two-tailed Student’s *t* test. A *P* value < 0.05 was considered statistically significant. All statements of variability are for standard error of the mean (SEM) unless noted otherwise.

## Results

### Human RPE phagocytosis decreases with aging

**Fig. 1:**
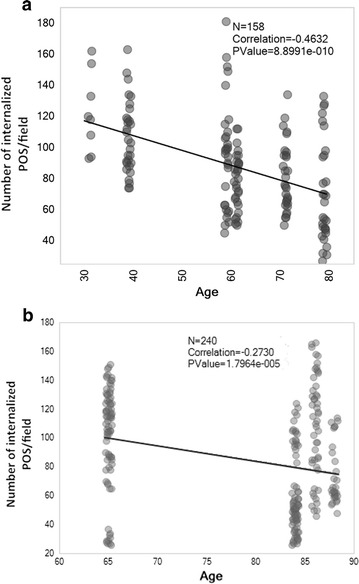
Phagocytosis level in RPE from human eyes of donors of different ages. RPE cells were isolated from eyes of 6 normal donors (no significant ocular medical history) with age 31, 39, 59, 61, 71 and 79, and 4 AMD donors with age 65, 84, 86, and 88 and cultured as described in “Methods”. For phagocytosis assay, 5 × 10^4^ human RPE cells were plated in a 24-well plate, maintained in MEM containing 20% (v/v) FBS for at least 6 days, then in MEM with 5% FBS (v/v) before the assay. Circle shown for normal age 31 represents 10 counted numbers of internalized OS from 10 representative fields from one experiment. The circles for all the other age groups represent counted numbers of internalized OS from 10 representative fields per experiment from three independent experiments for each age group. A moderate negative (**a**) and weak negative (**b**) correlation between phagocytosis level and age was shown in the normal and AMD RPE, respectively

To assess how the human RPE phagocytic function changes with aging, phagocytosis assay was performed in RPE cells isolated from eyes of six normal donors with no known ocular diseases aged 31–79 years old. We found that there is a moderate negative correlation between phagocytosis level and age (Pearson correlation = − 0.46 and P value = 8.9e−010, Fig. 1a) in normal human RPE cells. Changes in phagocytosis level was also assessed in RPE cells isolated from eyes of four AMD donors aged 65–88 years old. Due to the limit of resources, we did not have eyes of AMD donors aged 70 s. We found that there is a weak negative correlation between phagocytosis level and age (Pearson correlation = − 0.27 and P value = 1.7964e−005, Fig. 1b) in AMD RPE cells. Taken together, these findings demonstrate that the RPE phagocytosis decreases with aging.

### Human RPE phagocytosis decreases in AMD

**Fig. 2:**
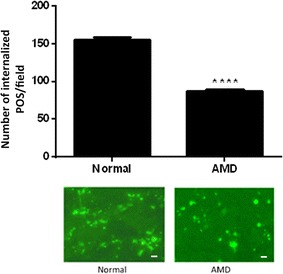
Phagocytosis level in RPE from eyes of AMD donors. RPE cells were isolated from eyes of 4 AMD donors with age 65, 84, 86, and 88, and cultured as described in “Methods”. For best possible age matching RPE isolated from 3 normal donors without ocular diseases of age 61, 71 and 79 were used as normal control. 5 × 10^4^ human RPE cells are plated in a 24-well plate in MEM containing 20% (v/v) FBS for a week followed by medium change to MEM with 5% (v/v) FBS and subjected to phagocytosis assay. *Normal* RPE cells from eyes of donors without ocular diseases, *AMD* RPE cells from eyes of donors with AMD. Data represent the mean ± SEM (n = 16). ****P < 0.0001. Representative images of RPE containing fluorescent ingested POS are shown (scale bar = 10 µm)

Among many theories proposed, circumstantial evidence suggests that oxidative stress and free radical damage in the RPE underlie the pathogenesis of AMD by impairing RPE phagocytic function [19, 24, 41]. However, there has been no definitive evidence of a phagocytic dysfunction in AMD to support this notion. Therefore, we isolated RPE cells from human eyes obtained from donors with AMD, and assessed their phagocytosis in comparison with those of RPE from best age-matched normal individuals. The age range for AMD donors is 65–88, and 61–79 for normal control donors with no known ocular diseases. We show that phagocytosis decreased significantly and dramatically in RPE of AMD donor eyes compared to that in age-matched normal RPE (Fig. 2). To the best of our knowledge this is the first evidence definitively and quantitatively showing the phagocytic function defect in human RPE from AMD donors.

### hUTC conditioned medium promotes phagocytosis in RPE from aged normal and AMD donors

**Fig. 3:**
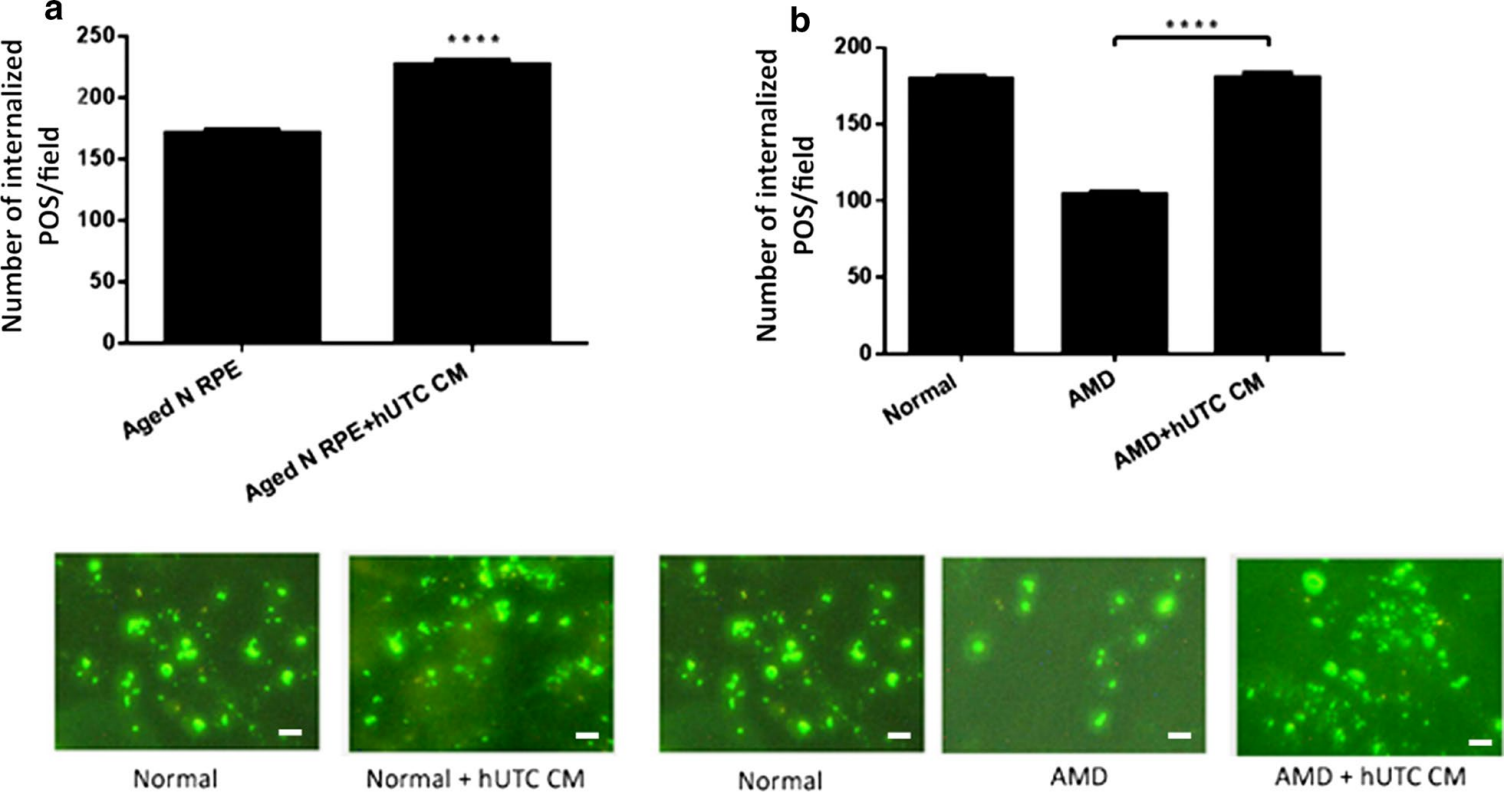
Effect of hUTC conditioned medium on phagocytosis in RPE from aged normal and AMD donors. hUTC conditioned medium (CM) was prepared as described in “Methods”. **a**, **b** RPE cells were isolated from eyes of 3 normal donors without ocular diseases at age 61, 71, and 79, and **b** from 4 AMD donors at age 65, 84, 86, and 88, respectively, and cultured before experiments. 5 × 10^4^ human RPE cells were plated in a 24-well plate in MEM containing 20% (v/v) FBS for a week. For untreated cells, medium was changed to MEM containing 5% (v/v) FBS. The cells to be treated were incubated with hUTC CM for 24 h and then subjected to phagocytosis assay in the presence of hUTC CM. *N* RPE cells from eyes of donors without ocular diseases, *AMD* RPE cells from eyes of donors with AMD, *CM* conditioned medium. Data represent the mean ± SEM (n = 26). ****P < 0.0001. Representative images of RPE containing fluorescent ingested POS are shown (scale bar = 10 µm)

To examine the effect of hUTC conditioned medium (CM) on phagocytosis in human RPE, we performed phagocytosis assay in RPE isolated from eyes of aged donors (age range 61–79) without ocular diseases and from donors with AMD (age range 65–88), both pretreated with hUTC CM for 24 h and then subjected to phagocytosis assay (Fig. 3a, b). We show that hUTC CM significantly promoted phagocytosis in RPE from aged normal eyes, and rescued the phagocytic dysfunction in the RPE from AMD eyes.

### Recombinant human RTK ligands and bridge molecules promote human RPE phagocytosis

**Fig. 4:**
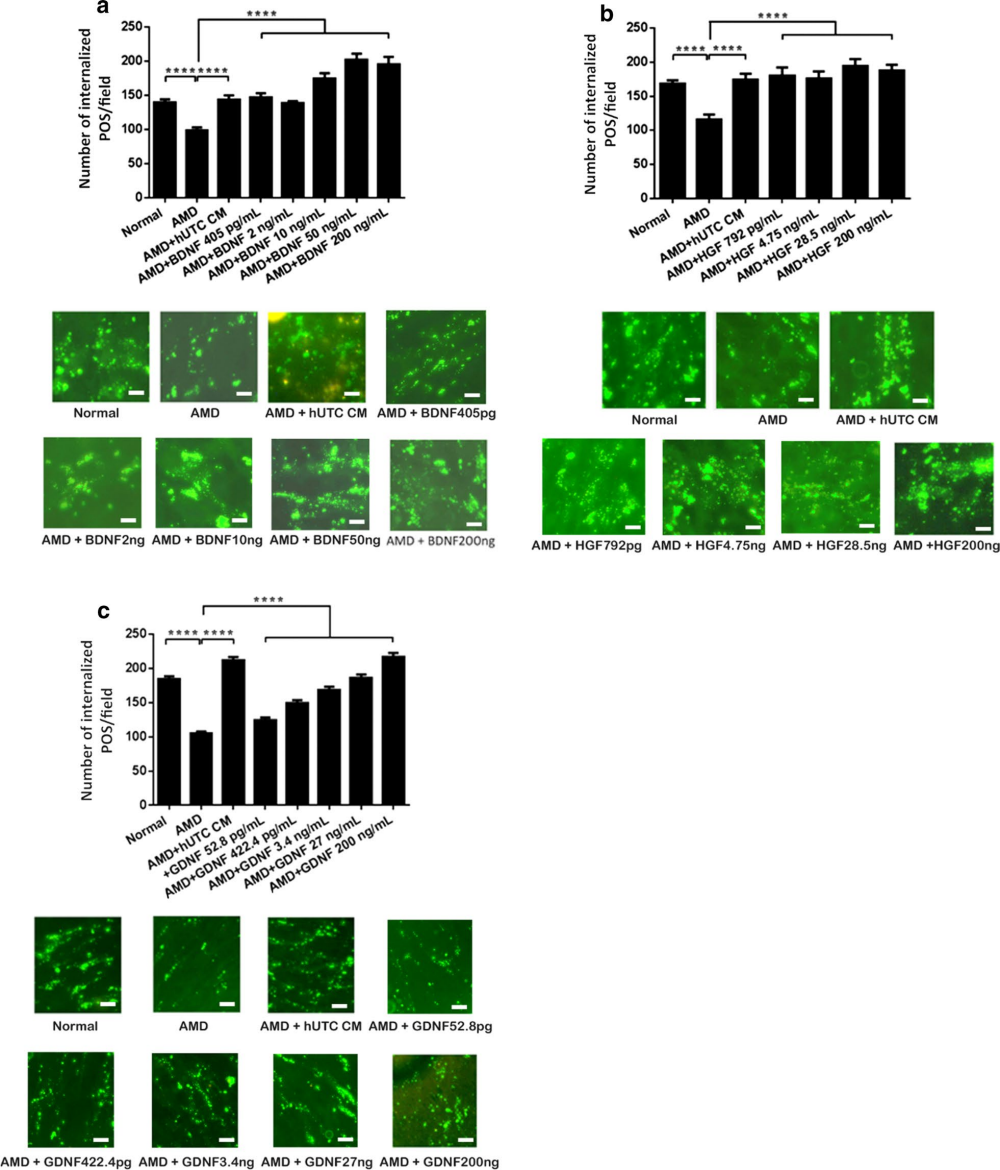
Effect of RTK ligands on phagocytosis in human RPE from donor eyes with AMD. The human RPE cells isolated from eyes of 4 AMD donors with age 65, 84, 86, and 88 were incubated with recombinant human BDNF (**a**), HGF (**b**), or GDNF (**c**) for 24 h, and then subjected to phagocytosis assay. The human RPE incubated with the hUTC CM was used as a positive control for the assay. *Normal* RPE cells from eyes of donors without ocular diseases, *AMD* RPE cells from eyes of donors with AMD, *CM* conditioned medium. Data represent the mean ± SEM (n = 4). ****P < 0.0001. Representative images of RPE containing fluorescent ingested POS are shown (scale bar = 10 µm)

**Fig. 5:**
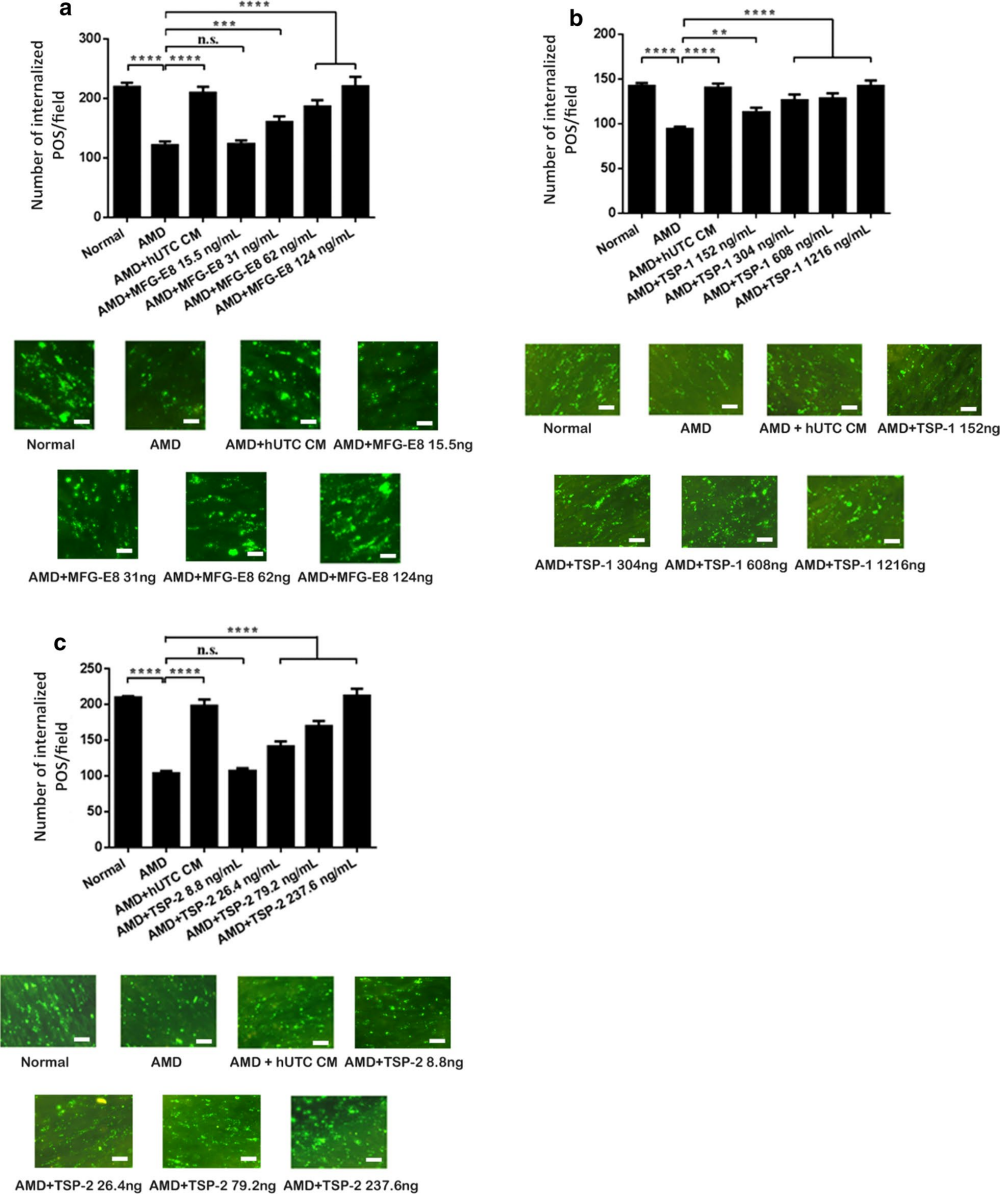
Effect of bridge molecules on phagocytosis in human RPE from donor eyes with AMD. The human RPE cells isolated from eyes of 4 AMD donors with age 65, 84, 86, and 88 were cultured as described in “Methods”. The photoreceptor OS were incubated with recombinant human MFG-E8 (**a**), TSP-1 (**b**), or TSP-2 (**c**) for 24 h and then fed to the RPE cells for phagocytosis assay in the absence of the hUTC CM. The POS pre-incubated with the hUTC CM was used as a positive control for the assay. *Normal* RPE cells from eyes of donors without ocular diseases, *AMD* RPE cells from eyes of donors with AMD, *CM* conditioned medium. Data represent the mean ± SEM (n = 4). ****P < 0.0001, ***P < 0.001, **P < 0.01, *ns* not significant. Representative images of RPE containing fluorescent ingested POS are shown (scale bar = 10 µm)

hUTC-secreted RTK ligands (BDNF, HGF, GDNF) and bridge molecules (MFG-E8, TSP-1, TSP-2) were shown to rescue phagocytosis in the RCS RPE [46]. Here, we investigated the effect of the RTK ligands and bridge molecules on phagocytosis in human RPE cells isolated from eyes of AMD donors. For the functional test of RTK ligands, we incubated the human RPE with recombinant human BDNF, HGF, and GDNF individually for 24 h and then performed phagocytosis assay with the addition of human POS. The RPE incubated with the hUTC CM was used as a positive control. For the functional test of bridge molecules, isolated human POS were incubated with each of the recombinant human MFG-E8, TSP-1, and TSP-2, respectively, for 24 h and then fed to the human RPE cells for phagocytosis assay. The POS incubated with the hUTC CM was used as a positive control. The minimum doses used for each RTK ligand and bridge molecule were the same as what they were found in the hUTC CM by ELISA [46]. HGF rescued AMD RPE phagocytosis at all the doses applied. BDNF, and GDNF dose-dependently increased the phagocytosis level in the AMD RPE cells. The effect of BDNF and HGF are the strongest even at the lowest dose. When applied at higher concentrations, BDNF was able to promote phagocytosis at a much higher level than that in normal control RPE (Fig. 4a–c). Similar dose–response effects were observed with the bridge molecule MFG-E8, TSP-1, and TSP-2 (Fig. 5a–c). These findings demonstrate that recombinant RTK ligand and bridge molecule proteins can mimic the effect of the hUTC CM and restore phagocytosis in AMD RPE cells, suggesting that these factors could be involved in the hUTC-mediated phagocytosis rescue in the AMD RPE.

### Effect of hUTC conditioned medium on gene expression in human RPE cells

**Fig. 6:**
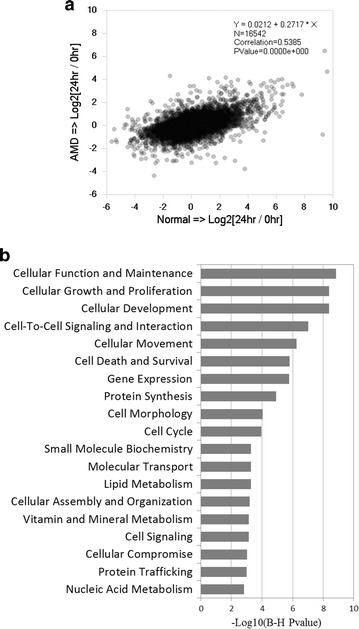
Effect of hUTC conditioned medium on gene expression in human RPE cells. **a** Gene expression changes after 24-h hUTC CM treatment in human normal (X axis) and AMD (Y axis) RPE cells. The changes in gene expression between the two cell types are significantly correlated (P value = 0). **b** Enrichment of genes modulated by hUTC CM treatment in molecular and cellular functions (adjusted P value < 0.05). 1811 genes significantly upregulated or downregulated (fold change > 2 and adjusted P value < 0.05) in response to hUTC CM treatment were enriched in 19 molecular and cellular functions

To examine the impact of hUTC on biological processes in RPE cells that may be associated with its phagocytosis-promoting effect, we proceeded to examine the effects of hUTC CM on gene expression in RPE cells isolated from normal and AMD donor eyes using RNA-Seq based transcriptomic profiling. After mapping sequence reads of all samples onto genome and quantifying gene expression, we selected 16542 expressed genes (TPM > 0.1 in any sample group) among all 27453 genes in human genome as defined by the refGene gene model, and applied them to downstream analysis. Gene expression changes after 24-h hUTC CM treatment in human normal and AMD RPE cells were analyzed by differential gene analysis. The changes in gene expression between the two cell types are significantly correlated (P value = 0, Fig. 6a). hUTC CM treatment demonstrated similar trend of effect on both normal and AMD RPE cells in up- or down-regulating gene expression (Pearson correlation = 0.54 and P value = 0, Fig. 6a). Most gene expression changes in AMD RPE cells are less prominent than those in normal RPE cells (AMD versus normal trend line slope = 0.27, Fig. 6a).

Combining samples from all normal and AMD donors, we identified 1811 genes significantly induced or suppressed (fold change > 2 and adjust P value < 0.05) by the hUTC CM treatment, and applied the genes to Gene Set Enrichment Analysis (GSEA) using Ingenuity Pathway Analysis (IPA) tool. These genes are significantly enriched (adjusted P value < 0.05) in 19 molecular and cellular functions (Fig. 6b). Using gene ontology classification, we further identified up-regulated genes with functions covering cellular movement regulation, inhibition of apoptosis, inflammation and oxidative stress, as well as lipid metabolism (Table 1). We also identified down-regulated genes involved in cellular movement regulation and apoptosis or inflammation induction (Table 2).

**Table 1:** Sorted genes upregulated by hUTC conditioned medium treatment in human RPE cells isolated from AMD and normal donors

IPA category	Function	Gene symbol	Gene name
Cellular movement	Receptor tyrosine kinase	MERTK	MER proto-oncogene, tyrosine kinase
	Rho GTPases related	PLD1	Phospholipase D1
		PAK3	p21 (RAC1) activated kinase 3
	Growth factors	GDNF	Glial cell derived neurotrophic factor
Cell death and survival	Anti-apoptosis	ADM	Adrenomedullin
		EPOR	Erythropoietin receptor
		SOCS3	Suppressor of cytokine signaling 3
		NR4A2	Nuclear receptor subfamily 4 group A member 2
		PIM1	Pim-1 proto-oncogene, serine/threonine kinase
		GDF9	Growth differentiation factor 9
		BIRC3	Baculoviral IAP repeat containing 3
		G0S2	G0/G1 switch 2
Cellular function and maintenance	Anti-inflammation	ESR2	Estrogen receptor 2
		NFKBIA	NFKB inhibitor alpha
		ZC3H12A	Zinc finger CCCH-type containing 12A
	Anti-oxidative stress	SOD2	Superoxide dismutase 2, mitochondrial
		ALB	Albumin
Lipid metabolism	Enzymes	ABCA1	ATP binding cassette subfamily A member 1

**Table 2:** Sorted genes downregulated by hUTC conditioned medium treatment in human RPE cells isolated from AMD and normal donors

IPA category	Function	Gene symbol	Gene name
Cellular movement	Rho GTPases related	ARHGAP9	Rho GTPase activating protein 9
Cell death and survival	Apoptosis induction	CYCS	Cytochrome c, somatic
		BMP5	Bone morphogenetic protein 5
		EGR3	Early growth response 3
Cellular function and maintenance	Inflammation induction	TLR4	Toll like receptor 4
		TAC1	Tachykinin precursor 1
		FABP4	Fatty acid binding protein 4
		CCRL2	C–C motif chemokine receptor like 2
		PTGDR	Prostaglandin D2 receptor
		F2RL1	F2R like trypsin receptor 1
		PLA2G7	Phospholipase A2 group VII

Rho GTPases play a central role in actin polymerization and cytoskeleton reorganization, the driving force of phagocytosis [49]. We found that *ARHGAP9*, a gene encoding Rho GTPase-activating protein 9, was downregulated by hUTC CM treatment, while *GDNF*, *MERTK* and two Rho GTPase effectors, *PLD1* and *PAK3*, were upregulated by the treatment. These findings suggest that hUTC-secreted factors may activate Rho GTPase pathway to promote phagocytosis in human RPE cells.

Among the inflammation associated genes regulated by hUTC CM, estrogen receptor beta 2 (*ESR2*), known to have a protective effect against matrix dysregulation and possibly deposit formation [50], was upregulated; *NFKBIA*, an inhibitor of NF-κB which is reported to play a role in signal transduction pathways of oxidative stress in RPE cells [51] was upregulated; and toll-like receptor 4 (*TLR4*), a mediator of innate immunity proposed to play a role in the pathogenesis of AMD [52] was downregulated by hUTC CM in human RPE, indicating generally a protective effect against oxidative, inflammatory, and immune stress.

Other genes we identified of specific interest that are upregulated by hUTC CM treatment include superoxide dismutase 2 (*SOD2*), an anti-oxidant enzyme that is important in oxidative stress response [53, 54], and ATP-binding cassette transporter 1 (*ABCA1*), a major regulator of cellular cholesterol and phospholipid homeostasis which functions as a cholesterol efflux pump [55]. These results indicate a protective role of hUTC-secreted factors against oxidative stress and cholesterol build-up in the RPE.

## Discussion

Despite ample evidence for the involvement of RPE phagocytosis in AMD, actual alteration in this function in AMD has not been demonstrated [15, 16, 19, 31, 41, 42, 56]. To the best of our knowledge, we have shown for the first time a decrease in the POS phagocytic function by RPE from AMD patients. An important question is whether this dysfunction is the result of AMD pathology or it is an early phenomenon that occurs before any significant AMD pathology which may actually play a role in the pathogenesis of AMD, i.e., cause or effect.

There are certainly enough reasons for this phagocytic dysfunction to be a result of AMD pathology. Lipofuscin, which is known to accumulate in the RPE in AMD patients, has been shown to be toxic to cells, including the phagocytic function [39, 57]. Lipofuscin can occupy as much as 19% of cellular space in the RPE of older individuals (81–90 years old), while it only accounts for ~ 1% of cellular space in young individuals (first decade), and it can reach massive amounts in AMD patients [27, 58, 59]. Its bis-retinoid A2E content inhibits phagocytosis, lysosomal activities, and mitochondrial function [3, 39, 40, 60]. Another cause of the phagocytic dysfunction could be the accumulation of iron which has been shown in the RPE in AMD [41, 61]. Excess iron produces hydroxyl radicals leading to oxidative stress, and has been shown to inhibit phagocytosis and cathepsin D processing, important for the lysosomal processing stage of phagocytosis [31, 42, 62, 63].

On the other hand, there are some indications that the phagocytic dysfunction can be more causal in the pathogenesis of AMD, that is, not necessarily the primary etiology but an early phenomenon that can play an important role in the pathogenesis of AMD. A notable indication observed in our data is that a significant phagocytic dysfunction (~ 50% of age-matched normal), far more than attributable to aging, was present in the RPE from a relatively young AMD patient (65 years old). Anomalies in any stage of the POS shedding and phagocytosis have been thought to cause various ocular disorders, including retinitis pigmentosa (RP) and AMD [64–67]. One of the earliest hypotheses of retinal degeneration, including AMD, was that accumulation of abnormal quantities of POS breakdown products in RPE lead to cellular dysfunction and degeneration [68]. A phagocytic dysfunction can certainly do this. There is ample evidence that a defect in POS phagocytosis by RPE leads to dire consequences for the retina. A classic example is the RCS rat model of retinal degeneration in which a defect in RPE phagocytosis due to a genetic defect in Mer tyrosine kinase causes photoreceptor death [43]. A knockout model of a phagocytosis receptor, alphavBeta5, in a mouse model results in phagocytic dysfunction and a phenotype resembling atrophic AMD [44]. A defect in phagocytosis can also be at the level of phagolysosomal processing to cause retinal problems as shown in a transgenic model of the lysosomal enzyme cathepsin D [45]. This model also demonstrated a phenotype resembling atrophic AMD.

So if phagocytic dysfunction is an early occurrence, not necessarily caused by AMD but causal in AMD, what can be causing it? Aside from AMD, a decrease in phagocytic function is observed with age [39, 69–71]. Two factors that can contribute to the decrease in phagocytic function with age are lipofuscin and oxidative stress, both of which have been shown to increase with age [14, 19, 58, 72–74]. The deleterious effect of A2E in lipofuscin on phagocytic function has been well documented [3, 15, 31, 75, 76], and oxidative stress has been demonstrated to decrease phagocytosis [77]. These two factors could begin to affect RPE phagocytosis negatively very early in the pathogenesis of AMD even before any real pathology is present.

A decrease or dysfunction in RPE phagocytosis has been shown to lead to lipid formation [3] and lipofuscinogenesis [15, 16, 36, 78], which in turn, inhibits phagocytosis. A direct link has been shown between abnormality in RPE phagocytosis and lipofuscin accumulation, postulated to lead to AMD, in animal models [44, 45]. If lipofuscin is derived from POS phagocytosis via incomplete digestion because of phagocytic dysfunction and lipofuscin in turn can inhibit phagocytosis, it is a self-perpetuating vicious cycle which may be an important mechanism in the pathogenesis of AMD. Accumulation of lipofuscin, aside from inhibiting phagocytosis, is toxic to cellular functions, including lysosomal and mitochondrial functions, and will lead to RPE death. Meanwhile, POS phagocytosis by RPE is thought to cause oxidative stress through production of reactive oxygen species [5, 19, 79, 80], and oxidative stress inhibits phagocytosis [77], so this is also a self-perpetuating suicidal process which links to further phagocytic dysfunction and lipofuscin accumulation. Thus, the negative interaction between phagocytosis, lipofuscin, and oxidative stress can be a mutually promoting, downward spiral that may be an early mechanism in the pathogenesis of AMD, leading to RPE death and AMD.

The moderate decrease in phagocytic function with age was consistent with what has been reported in animals [69]. What is significant is that the decrease in POS phagocytosis by RPE seen in AMD patients is over and above the decrease seen with age, as shown by the difference seen between age-matched normal and AMD RPE. This was true in both the comparison of averages and comparisons of individual age-matched samples. In agreement, approximation of phagocytic levels in our best age-matched normal and AMD groups using the regression data from our age vs. phagocytosis experiment indicated that the decrease in phagocytosis attributable to the age difference in the two groups is only 13%, clearly less than the ~ 50% decrease we see in the AMD patients. Thus, the trend of decrease in RPE phagocytosis with age in normal donors is much less than the trend of RPE phagocytosis decrease observed in AMD donors. According to the hypothesis on mutually-promoting negative interaction presented above, this would suggest that, over and above the normal age-related decrease in RPE phagocytosis, individuals who develop AMD may have specific susceptibilities that result in aggravation of the interaction between phagocytosis, lipofuscin accumulation, and oxidative stress to result in a downward spiral.

The beneficial effect of hUTC in rescuing the phagocytic dysfunction in the AMD RPE is quite remarkable. hUTC was shown to rescue the phagocytic defect in the RPE in and from the RCS rat and ameliorate the associated retinal degeneration [46]. The phagocytic defect in the RCS rat is due to a genetic defect in the major phagocytic receptor, MerTK, and this defect results in retinal degeneration in the rat [43]. The same genetic defect has been shown in some cases of human retinal degeneration [81–88]. We had also shown that this phagocytosis rescue is mediated through the RTK ligands (BDNF, HGF, and GDNF), as well as bridge molecules (MFG-E8, TSP-1, and TSP-2) secreted by hUTC [46]. That hUTC is able to normalize a phagocytic dysfunction caused by a genetic defect in the MerTK gene in an animal model and a phagocytic dysfunction of unknown cause in the human RPE from AMD patients is significant. The phagocytic rescue by hUTC in the AMD RPE is apparently mediated, at least in part, by the same mechanisms involving the RTK ligands and bridge molecules as in the RCS RPE. These results speak to the great potency of hUTC in rescuing phagocytic defects in retinal degenerative conditions through trophic mechanism. If the phagocytic dysfunction we demonstrated in AMD RPE is related to the pathogenic mechanism of AMD as we have shown above, there may be a potential of hUTC for treatment of AMD.

In terms of the molecular mechanisms by which hUTC rescues phagocytosis, the results of the RNA-Seq analysis showed that by and large hUTC had similar effects on the overall gene expression in normal and AMD RPE. Among the genes affected by the hUTC CM treatment which are enriched in 19 molecular and cellular functions, we further identified up-regulated genes with functions covering cellular movement regulation, inhibition of apoptosis, inflammation and oxidative stress, as well as lipid metabolism. We also identified down-regulated genes involved in cellular movement regulation and apoptosis or inflammation induction. *GDNF*, *MERTK* and two Rho GTPase effectors, *PLD1* and *PAK3*, were upregulated by the treatment, suggesting that hUTC-secreted RTK ligands, such as GDNF, BDNF and HGF, and possibly other RTK ligands and factors, could enhance Rho GTPase signaling pathway and its downstream effectors, thereby promoting phagocytosis in human RPE cells. It has been shown that the underlying function of MerTK in rod outer segment ingestion by the RPE is regulation of the cytoskeleton reorganization apparently through activation of phosphoinositide 3 (PI3) kinase and Rho GTPases [89–91]. Rho GTPases play a central role in plasma membrane and cytoskeleton remodeling during particle internalization by interacting with and activating downstream effectors, such as PLD1 and PAK3, to control the assembly and organization of actin filaments [49, 92, 93]. Stimulation of RTKs by RTK ligands, such as BDNF, GDNF and HGF, can induce Rho GTPase activation [94]. Rho GTPases cycle between GDP-bound inactive form and GTP-bound active state that are regulated by guanine-nucleotide exchange factors (GEFs) and GTPase-activating proteins (GAPs), respectively. GEFs stimulate the exchange of GDP for GTP to generate the activated state, whereas GAPs increase the intrinsic Rho GTPase activity to accelerate the return of the proteins to the inactive state [95, 96]. *ARHGAP9*, a gene that encodes Rho GTPase-activating protein 9, was downregulated by hUTC CM treatment.

Among the inflammation associated genes regulated by hUTC CM, *ESR2, NFKBIA* and *TLR4* are of particular interest. In vivo murine models of estrogen receptor beta (ESR2) knockout have been associated with altered murine matrix metalloprotease-2 activity, increased collagen production, and sub-RPE deposit formation as seen in human dry AMD [50]. Upregulation of *ESR2* by hUTC CM in human RPE may have a protective effect against matrix dysregulation, thereby reducing deposit formation and improving RPE health. Nuclear factor kappaB (NF-κB) was reported to play a role in signal transduction pathways of oxidative stress in RPE cells [51]. NF-κB inhibitor alpha (NFKBIA) inhibits NF-κB by masking the nuclear localization signals of NF-κB proteins and keeping them sequestered in an inactive state in the cytoplasm [97]. Upregulation of *NFKBIA* gene by hUTC CM could potentially enhance the inhibition of the NF-kB pathway and protect RPE from oxidative stress. Toll-like receptor 4 (TLR4) is a mediator of innate immunity which is proposed to play a role in the pathogenesis of AMD associated with retinal inflammation and cell death [52]. We found that *TLR4* was downregulated by hUTC CM in human RPE, indicating a protective effect against TLR4-mediated immune response.

Other genes we identified of specific interest that are upregulated by hUTC CM treatment are *SOD2* and *ABCA1*. The upregulation of SOD2 and ABCA1 by hUTC CM treatment indicates a protective role of hUTC-secreted factors against oxidative stress and cholesterol build-up in the eye. The SOD2 is an anti-oxidant enzyme that functions to clear mitochondrial reactive oxygen species and confer protection against cell death in response to oxidative stress and inflammation. It was reported that knockdown of *Sod2* in the RPE in a mouse model of geographic atrophy (GA) leads to elevated oxidative stress, causing some of the features of GA including damage to the RPE and death of photoreceptors [53, 54]. Depletion of SOD2 in the RPE in mice shows accumulation of lipofuscin-like fluorescent aggregates containing A2E [98]. ATP-binding cassette transporter ABCA1 is a protein which in humans is encoded by the *ABCA1* gene. This transporter is a major regulator of cellular cholesterol and phospholipid homeostasis, and functions as a cholesterol efflux pump in the cellular lipid removal pathway [55]. Downregulation of ABCA1 in senescent macrophages disrupts the cell’s ability to remove cholesterol from its cytoplasm, leading the cells to promote the pathologic atherogenesis which plays a central role in common age-associated diseases such as atherosclerosis and AMD. In AMD, cholesterol is known to accumulate in the eye in drusen [99, 100]. Knockout mouse models of AMD treated with agonists that increase ABCA1 in loss of function and gain of function experiments demonstrated the protective role of elevating ABCA1 in regulating angiogenesis in eye disease [101].

Thus, the factors in hUTC CM affected a variety of genes in human RPE cells that are involved in a wide range of biological processes, including phagocytosis and signaling pathways, regulation of apoptosis, inflammation, oxidative stress and metabolism. All of these could contribute to the phagocytosis rescue effect mediated by hUTC CM in the human RPE.

## Conclusions

RPE phagocytosis has often been implicated in the pathogenic mechanism of AMD, but an alteration in this function has not been directly demonstrated in AMD. To the best of our knowledge, we show a dysfunction in RPE phagocytosis directly in the RPE from AMD patients for the first time in this report. There is ample evidence to implicate phagocytosis in the pathogenesis of AMD because of its relationship to lipofuscin, drusen, and oxidative stress. In this report, however, we present evidence and argument that RPE phagocytic dysfunction may be involved early in the genesis of AMD, rather than late as an effect of AMD. Remarkably we also show that hUTC is able to correct this phagocytic dysfunction in the AMD RPE, just as it did in the RCS rat RPE and ameliorated the retinal degeneration. If RPE phagocytic dysfunction is causally involved in the mechanism of AMD as we postulate, hUTC may prove to be beneficial for the treatment of AMD. In summary, our findings provide insights into the understanding of AMD pathogenesis and demonstrate the potential of hUTC for the treatment of AMD.

## Additional file

**Additional file 1: Figure S1.** Immunofluorescence of human RPE culture. Human RPE cells were obtained from human donor eyes and cultured as described in “Methods”. The RPE culture in 24-well plate was stained with the Pan-Keratin (C11) monoclonal antibody, the Alexa Green-conjugated secondary antibody, and examined by fluorescence microscopy as described in “Methods”. **(A)** RPE cells stained with the Pan-Keratin antibody, **(B)** RPE cells stained only with the secondary antibody. The left panel shows the immunofluorescence, and the right shows the phase images. The RPE culture is positive for Pan-Keratin, including CK8 and CK18, confirming it as RPE (47). The staining with the secondary antibody only is negative. Scale bar = 10 µm.

## Abbreviations

ABCA1: ATP-binding cassette transporter 1
AMD: age-related macular degeneration
ARHGAP9: Rho GTPase activating protein 9
ATCC: American Type Culture Collection
ATP: adenosine triphosphate
BDNF: brain-derived neurotrophic factor
CM: conditioned media
DMEM: Dulbecco’s modified Eagle’s medium
DNA: deoxyribonucleic acid
ELISA: enzyme-linked immunosorbent assay
ESR2: estrogen receptor beta 2
FBS: fetal bovine serum
FITC: fluorescein isothiocyanate
GA: geographic atrophy
GAP: GTPase-activating proteins
GDNF: glial cell-derived neurotrophic factor
GDP: guanosine diphosphate
GEF: guanine-nucleotide exchange factor
GSEA: Gene Set Enrichment Analysis
GTP: guanosine triphosphate
HBSS: Hank’s balanced salt solution
HEPES: 4-(2-hydroxyethyl)-1-piperazineethanesulfonic acid
HGF: hepatocyte growth factor
hUTC: human umbilical tissue derived cells
IPA: Ingenuity Pathway Analysis
MEM: minimum essential medium
MERTK: MER proto-oncogene, tyrosine kinase
MFG-E8: milk-fat-globule-EGF-factor 8
NDRI: national disease research interchange
NFKBIA: NFKB inhibitor alpha
NF-κB: nuclear factor kappa-light-chain-enhancer of activated B cells
PAK3: P21 (RAC1) Activated Kinase 3
PBS: phosphate buffered saline
PI3: phosphoinositide 3
PLD1: phospholipase D1
POS: photoreceptor outer segments
RCS: Royal College of Surgeons
RNA: ribonucleic acid
RPE: retinal pigment epithelium
RTK: receptor tyrosine kinase
SEM: standard error of the mean
SOD2: superoxide dismutase 2
TLR4: toll-like receptor 4
TPM: transcripts per million
TSP-1: thrombospondin 1
TSP-2: thrombospondin 2
VEGF: vascular endothelial growth factor
