# Supplementary material for: RPE phagocytic function declines in age-related macular degeneration and is rescued by human umbilical tissue derived cells
Source: J Transl Med. 2018 Mar 13;16:63. doi: 10.1186/s12967-018-1434-6 (PMC5851074; doi:10.1186/s12967-018-1434-6)
Supplement: Supplementary file 1 — Additional file 1: Figure S1. Immunofluorescence of human RPE culture. Human RPE cells were obtained from human donor eyes and cultured as described in “Methods”. The RPE culture in 24-well plate was stained with the Pan-Keratin (C11) monoclonal antibody, the Alexa Green-conjugated secondary antibody, and examined by fluorescence microscopy as described in “Methods”. (A) RPE cells stained with the Pan-Keratin antibody, (B) RPE cells stained only with the secondary antibody. The left panel shows the immunofluorescence, and the right shows the phase images. The RPE culture is positive for Pan-Keratin, including CK8 and CK18, confirming it as RPE (47). The staining with the secondary antibody only is negative. Scale bar = 10 µm. [file 12967_2018_1434_MOESM1_ESM.docx]

**Fig. S1**

**
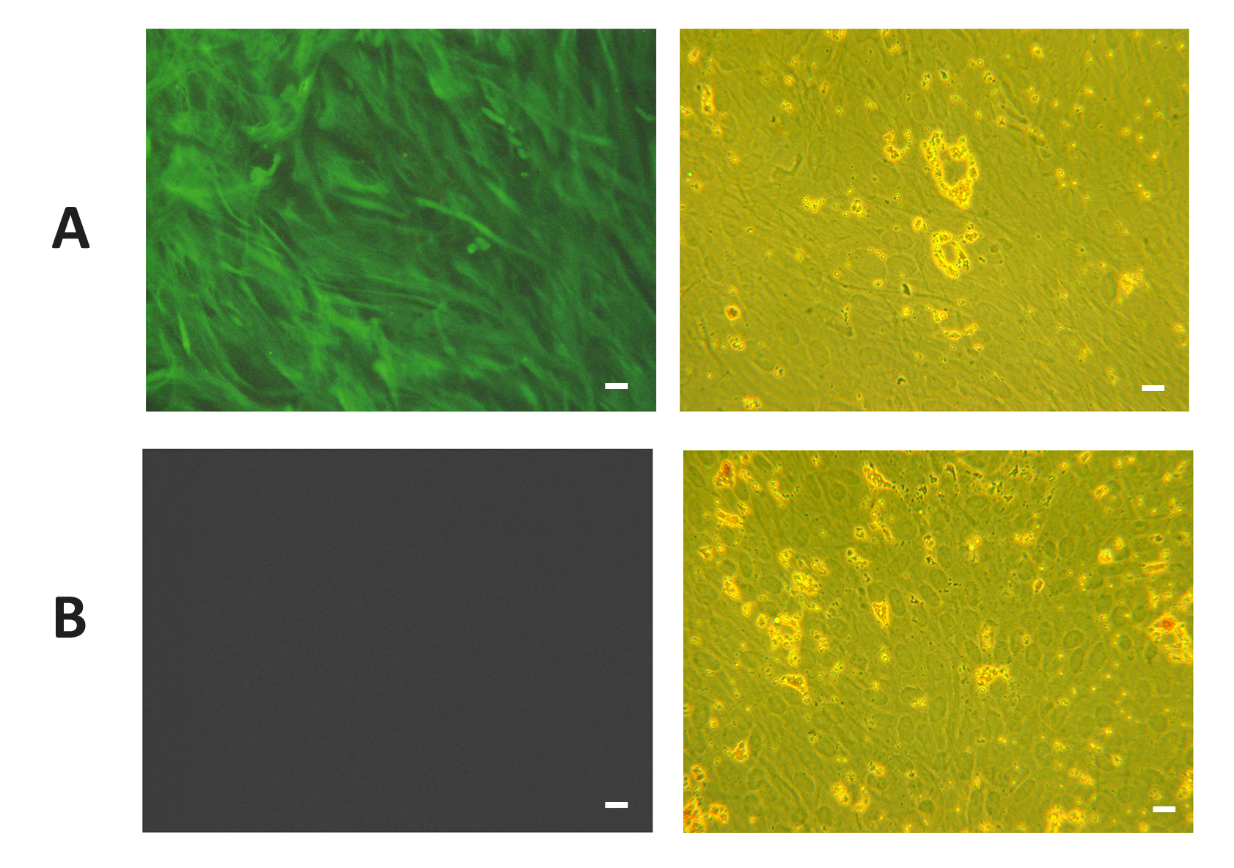
**

**Figure S1. Immunofluorescence of human RPE culture.**

Human RPE cells were obtained from human donor eyes and cultured as described in Methods. The RPE culture in 24-well plate was stained with the Pan-keratin (C11) monoclonal antibody, the Alexa Green-conjugated secondary antibody, and examined by fluorescence microscopy as described in Methods. **(A)** RPE cells stained with the Pan-keratin antibody, **(B)** RPE cells stained only with the secondary antibody. The left panel shows the immunofluorescence, and the right shows the phase images. The RPE culture is positive for pan-keratin, including CK8 and CK18, confirming it as RPE (47). The staining with the secondary antibody only is negative. Scale bar = 10µm.
